# Anticipating Change: Local Attitudes Toward a New Community Emergency Medicine Residency Program

**DOI:** 10.5811/westjem.65682

**Published:** 2026-11-02

**Authors:** J. Austin Lee, Ben Johnson, Joseph T. Cornelius

**Affiliations:** * Indiana University School of Medicine, Department of Emergency Medicine, Bloomington, Indiana; † Indiana University School of Medicine, Bloomington, Indiana; ‡ Indiana University School of Medicine, Department of Medical Sciences, Bloomington, Indiana

## Abstract

**Introduction:**

The Indiana University School of Medicine launched two new community-based residency programs at Indiana University Health Bloomington (IUH-B) in July 2026. Prior to the launch, this research explored community members’ attitudes, expectations, and concerns regarding the introduction of resident physicians to the emergency department (ED).

**Methods:**

This study was conducted from June to July 2025 at IUH-B. Using a convergent mixed-methods design, we evaluated a convenience sample of adult patients and accompanying family members in the emergency department (ED). Data collection included a 16-item electronic survey and semi-structured interviews.

**Results:**

The survey was completed by 39 adult participants, and 40 completed the qualitative interview. Most participants (59%) were aware of the residency programs, and nearly all (95%) agreed that residents would improve the quality, access, and modernity of healthcare in Bloomington, with very few expressing concerns about resident involvement. Overall, 97% of respondents stated support for the expansion of additional physician training programs locally. Comfort with or support for residency programs was not significantly influenced by prior experience with residents or awareness of the new programs. Thematic analysis shows that participants value residents who are competent, personable, and attentive listeners, while emphasizing the importance of strong supervision by attending physicians. Overall, participants are optimistic that new residency programs will bring more clinicians and up-to-date medical practices to the Bloomington area.

**Conclusion:**

Participants viewed the implementation of a new residency program positively, agreeing with proposed benefits and expressing minimal concerns. To ensure successful integration, further community initiatives should focus on educational outreach regarding the specific roles and training levels of emergency medicine residents, and prepare for implementation of trainees.

## INTRODUCTION

In July 2026, the Indiana University School of Medicine launched two physician residency training programs in Bloomington, Indiana, one of which is an emergency medicine (EM) residency. Prior research suggests that the expansion of residency programs enhances the quality of patient care, supports long-term workforce development through improved recruitment and retention, increases access to care, and expands local and regional opportunities for medical education. 1–4 Patient care and community support for new medical training programs, depend heavily on how patient and family members perceive resident-delivered care and develop trust in those trainees. 5–8

Prior studies have examined patient and community members’ perspectives of resident physicians working in clinical and surgical practice environments in family medicine, orthopedics, and neurosurgery; however, no studies have assessed these perspectives in the field of emergency medicine. 5,9–13 While a study of the implementation of a general surgery program in rural Appalachia found strong local support for the presumed community benefits and improved access to care,^5^ other studies have found that community members misunderstand the roles, responsibilities, and levels of physician education and training. 8,9,14,15

This study explores community members’ knowledge, expectations, and concerns regarding the introduction of resident physicians in the emergency department (ED) clinical setting at Indiana University Health Bloomington (IUH-B). The goal was to acquire insights that would inform program planning, patient communication, and community engagement strategies ahead of the program launch.

## METHODS

### Study Setting and Design

The IUH-B is a Level III trauma center in Bloomington, Indiana with 60 emergency department (ED) beds and more than 53,000 visits annually. 16 We collected data from patients and family members during their ED visit from June 25 to July 10, 2025. The quantitative survey and qualitative interview were performed at the same encounter; each participant was interviewed separately. Prior to this study, there had been limited outreach or publicity regarding program approval and implementation timeline. This study was reviewed and approved by the Indiana University Institutional Review Board (#27539).

We used a concurrent convergent mixed-methods design, incorporating structured electronic surveys and semi-structured interviews with adult patients and accompanying family members who received care in the IUH-B ED. Quantitative and qualitative data were integrated to triangulate findings (Figure 1). 17–19

We developed the survey instrument and semi-structured interview guide based on literature assessing patient and community attitudes toward resident physicians. The tools were directly informed by a survey used by Suliman et al in an evaluation of community stakeholder perceptions regarding a new rural general surgery residency program.^5^ Items were adapted and supplemented with additional questions specific to EN and the IUH-B community setting. Pilot testing was performed among the authors.

**Population Health Research Capsule**

What do we already know about this issue?

*Prior studies show graduate medical education in a community setting may improve care, workforce, and access.*

What was the research question?

*How do community members view the introduction of emergency medicine (EM) residents into a community emergency department?*

What was the major finding of the study?

*Community members surveyed strongly supported (95–97%) the new EM residency, citing expected benefits with few concerns.*

How does this improve population health?

*Community support and concerns can guide education, communication, and resident training as new EM programs are implemented.*

The electronic survey assessed general awareness of the incoming trainees, perceived benefits and concerns, and openness to receiving care from residents. Subsequent interviews (BJ) enabled us to better understand the context as themes of trust, continuity of care, and expectations were explored regarding trainee involvement in clinical encounters. If a participant had given an inaccurate definition of a medical resident in response to a survey question, the interviewer gave them the correct definition. The research team was led by JAL, an emergency physician with a background in public health and research. All three research group members are male. BJ is a medical student; JTC, a PhD candidate in the school of medicine, has performed in-depth work in qualitative methods and research.

### Selection of Participants

We obtained a convenience sample of participants among persons in the IUH-B ED clinical care spaces. Participants were excluded if they were less than 18 years of age, lacked consent capacity due to critical illness or incapacity, or were prisoners. Individuals were surveyed and interviewed in a private patient care space, or they were offered a private area for data collection if they were in a care area with limited privacy. Participants were not compensated for their time.

### Data Collection and Management

All participants received standard care at the discretion of their treating clinicians in the ED. Participants interacted with study personnel and completed the electronic survey (Appendix 1) and semi-structured interview (Appendix 2) in a single encounter. Refusal to participate was not tracked. Some participants were alone, while others had family or friends with them in their ED care space at the time of the survey/interview. Participants were given a password-protected tablet, and under the supervision of a study team member, they completed a 16-item electronic survey comprised of predominantly Likert-scale and yes/no questions (Appendix 1). Each survey and interview lasted 20 to 30 minutes per participant. No personally identifiable information was captured in the electronic survey, although participants’ voices were recorded anonymously during the interview. No demographic data such as age, sex, or gender identity were collected. Survey results were stored in a secure REDCap site, and all audio recordings, as well as transcripts and thematic coding, were saved in a password-protected, encrypted repository. 20,21

### Outcomes and Analysis

Both the survey and semi-structured interviews addressed five domains of incoming EM resident training at IUH-B: awareness and understanding, perceived benefits, concerns, trust, and looking to the future. Most of the survey questions were based on a five-point Likert scale. We used descriptive statistics and chi-square testing to analyze the quantitative findings. Qualitative results were examined using an iterative Saldana thematic analysis framework (Figure 2). 22

The semi-structured interviews were fully transcribed verbatim. BJ and JTC independently analyzed the and assigned codes to the data; they developed a codebook following multiple revisions. The first two versions of the codebook and sample coding were compared independently by each study author. Discrepancies were resolved through discussion. Qualitative interview responses were then assigned to categories and then designated to overarching themes. Codes were assigned to individual fragments of data. The coded responses were then categorized based on relationships, differences, similarities, and interactions. Finally, broad themes were analytically assigned to overlapping categories. We used concurrent convergent-mixed methods to compare and contrast the qualitative and quantitative findings.

## RESULTS

A total of 39 adult participants completed the electronic survey, and 40 completed the qualitative interview. One participant was willing to engage in the qualitative interview but declined to complete the electronic quantitative survey.

### Quantitative Results

The full compendium of quantitative results is available in Appendix 3. Twenty-three participants (59%) reported being aware of the upcoming residency programs at IUH-B, and nearly half (49%) correctly identified an emergency medicine resident as a medical school graduate in supervised specialty training. Although EM residents were not yet part of the IUH-B community at the time of the study, 20 respondents (51%) had previously been cared for by a resident physician in another setting, and none expressed dissatisfaction with that experience.

When asked about the potential benefits of having resident physicians in Bloomington, participants cited a shortage of local clinicians and agreed that residents would enhance IUH-B by improving healthcare quality, and access, with the the incorporation of up-to-date medical knowledge and practices. Two participants (5%) expressed concern that resident involvement could negatively affect their care; one individual (3%) voiced discomfort with residents’ training level; however, they did not correctly identify residents’ educational background.

Most participants agreed that they would feel trust and comfort if they were to receive care from EM residents, and nearly all trusted IUH-B to ensure safe, effective supervision. A total of 44% preferred that supervising physicians not be present for most resident interactions, suggesting confidence in residents’ competence. The majority (95%; *n* = 37) agreed that residency programs would improve healthcare in the Bloomington community, and 97% (*n* = 38) supported the expansion of additional training programs locally. Prior experience with residents or awareness of the new programs did not significantly influence comfort levels or support for residency expansion (**χ**^2^ = 0.004, *P* = .95; **χ**^2^ = 0.034, *P* = .85).

### Qualitative Results

To further understand how community members perceived the changes in the residency programs at IUH-B, we explored interview data via thematic analysis. Based on this iterative framework, a set of codes, broader subthemes, and themes were derived from the data. These domains included structured questions and topics to initiate the discussion; additionally, some unstructured discussion also followed (Appendix 2). Using interrater reliability, we found that the initial round of coding resulted in a Cohen kappa value of 0.724 (*P* < .001), indicating high interrater reliability.

The final codebook had four overarching themes (Table): benefits of residency programs; concerns regarding residents; trust (or distrust); and advice to residents from a patient perspective. Each theme is a synthesis of various categories, and categories contained one or more specific codes taken from interview responses.

### Themes and Subthemes

#### 1. Benefits of Residency Programs

Interview participants provided positive insights regarding new residency programs at IUH-B. System-level benefits included extra staff, increased clinicians, reduced wait times, increased retention, and up-to-date training. Community level benefits covered economic benefit, trust, and increased educational opportunities. One observed that “It could only get better... the more people you have out there working toward that goal the better.”

Participants also highlighted expanding research and educational opportunities locally, strengthening ties between the hospital and surrounding area.

[The new residency program] will attract more physicians to the area... expanding research, education, and knowledge in the area... building trust and friendship in the community.

#### 2. Concerns Regarding Residents

While participants expressed strong support, concerns were articulated about the training, confidence, and inexperience of resident doctors, highlighting the tension between teaching and providing top-notch service.

[I would be concerned] if they’re not knowledgeable... not taking their time... better ask somebody if they don’t know something.

Participants emphasized that proper supervision is needed, expressing fear that without it, mistakes could yield patient injury or improper care.

[I would be concerned about] someone who’s not as qualified... but if they do a good job overseeing them, I would be less concerned.

#### 3. Trust

Trust emerged as a central characteristic when discussing the future residency program, described as a relational process centered around patient-clinician communication and clinical demeanor.

Professionalism and respect would make me trust them.

Bedside manner is important... the way they listen to you.

Establishing clear communication and trust is essential to patient wellbeing and is a transactional process focused on residents’ professional development and patient satisfaction.

#### 4. Advice to Residents from Patients and Family

Participants were prompted to provide one piece of advice to incoming residents, frequently discussing the importance of collaboration, preparedness, and recognizing knowledge gaps.

Listen to patients and answer questions asked rather than blowing them off… connect with your team and think through things as a team.

Listen to patients, have confidence, and be humble… you don’t have to know it all.

Overall, participants were optimistic about the program, agreeing it would benefit the health system and community, with minimal concerns provided that residents remained under proper supervision and maintain professional communication.

## DISCUSSION

Approximately half of the surveyed individuals correctly identified the role of a resident physician—the most consistent finding between this study and others across multiple medical specialties. The percentage of people who can correctly identify the roles and responsibilities of residents is close to 50%; however, some studies found accurate identification to be as little as 20% to 30%.^6^,^8^,^9^,^12^–^15^ Notably, this knowledge gap did not appear to undermine trust; qualitative data showed that participants grounded their confidence in residents primarily in relational qualities such as communication and bedside manner, rather than in an accurate understanding of training level. Educational outreach may be needed to better inform the community about the role and training level of EM residents. In addition, defining roles while caring for patients in the ED should be emphasized in the training of residents.

More than half of those surveyed reported they were aware of the new EM residency program. While this finding was relatively higher than expected, there remains opportunity to improve communication with the community. Although the local press had reported on the program while we were administering the surveys, the news had not yet been widely reported at the time.^23^,^24^

Participants largely viewed the new residency program as a positive change within the community that would bring many benefits. Recurring themes were that implementation would lead to an increased number of clinicians, increased retention of those clinicians, and decreased wait times, as well as bring cutting-edge medicine, economic benefit, and community benefit as a whole to Bloomington. These qualitative themes were corroborated quantitatively, with more than 90% of survey respondents agreeing that the program would improve access, quality of care, and the incorporation of up-to-date medical practices. This suggests strong convergence between the two methods on the perceived benefits of the program, even though the interviews identified benefits (physician retention, economic benefit) that the structured survey did not directly explore. This aligns with prior research, with most patients agreeing that the expansion of graduate medical education programs is beneficial to their respective communities.^2^,^5^,^6^,^10^

A few concerns have been expressed about the program. Similar to what has been reported in other medical specialties, concerns include residents’ level of training (ie, inexperience) and the extend of proper supervision.^6^,^9^,^10^ While only 5% of survey respondents expressed hesitations about resident involvement, “concerns regarding residents” emerged as a full thematic category in interview data, suggesting open-ended discussion highlighted issues that our forced-choice survey items, and their positively anchored wording, did not fully capture. In this study and in others, most participants reported feeling comfortable with being treated by residents, and their confidence in residents improves if they know that the resident is in fact under supervision.^5^,^6^,^9^,^10^

The qualitative emphasis on supervision as a condition for trust contrasts with the 44% of survey respondents who preferred a supervising physician not be present for most resident interactions; together, these findings suggest patients may care less about an attending’s physical presence at every encounter than about knowing a supervisory structure exists as a backstop. The presence of an attending physician is likely of greater importance in high-stakes and procedural environments such as the operating room. This strengthens the argument that community education is needed to clarify the roles and responsibilities of residents and other members of the healthcare team.

Patients seem willing to trust resident physicians with their care and are more willing to trust those who demonstrate effective patient-clinician communication and display a professional clinical demeanor. These themes were brought up most frequently by the participants in this study, and they add to the sparse literature on these topics.

When given the opportunity to advise incoming residents, participants emphasized that residents should be collaborative with the healthcare team, prepared for the next phase of training, and willing to learn. Previous research suggests that patients believe it is most important that residents stay current with advances in medical knowledge.^2^,^6^ To improve patient trust, and therefore patient care, the residents’ didactic curriculum will need to emphasize professionalism and effective communication and collaboration between patients and the healthcare team.

## LIMITATIONS

The convenience sample was collected over a two-week period from a single ED, which may limit the generalizability of findings to other community hospital or academic settings. Demographic data such as age and sex were not collected, preventing analysis of how these variables may influence attitudes toward resident physicians. Although quantitative questions did include responses with negative and positive Likert-scale options, generally positive wording of survey questions may have introduced acquiescence bias. The sample size, while sufficient for exploratory mixed-methods work, is relatively small for drawing broad quantitative conclusions. Semi-structured interviews are also susceptible to interviewer-introduced bias and social desirability bias, because participants speaking face-to-face with an interviewer affiliated with the institution may have tempered negative opinions. Additionally, participants were recruited during an active ED visit, which may have introduced some response or selection bias and is not likely to be fully representative of true “community” knowledge or attitudes. Finally, as awareness of the incoming residency program was still limited at the time of data collection, future assessments following community education efforts may yield different results.

## CONCLUSION

Members of the Indiana University Health Bloomington community generally hold positive views and are broadly supportive of a new EM residency program. Most participants agreed that the new program would bring increased access to care, improved healthcare quality, and use of the most current medical practices and procedures, and they agreed this would improve the hospital overall. Some participants were not aware of the incoming residency programs, and as in prior work, community members often could not correctly distinguish a resident physician from other roles, such as a medical student. Because of this, further education is needed to ensure that the patients and family members understand the training level of their clinicians.

The community demonstrated little concern for the introduction of residents into their care. A few noted that they might be concerned with inexperience or arrogance but emphasized that proper oversight by more experiences clinicians would minimize these concerns. Furthermore, participants expressed trust in the healthcare team at IUH-B, including residents, to provide quality healthcare following implementation of the new program. In fact, the community supports the expansion of other residency programs in the area and agrees that this expansion would improve health care for Bloomington, Indiana, and the surrounding areas. Beyond being the first study (to our knowledge) to examine community attitudes toward a new emergency medicine residency, our findings identify communication and demeanor (rather than clinical competence alone) as the driver of patient trust, which can offer actionable targets for preimplementing community education as well as communication training for residents working in the emergency department and hospital setting.

## Supplementary Information







## Figures and Tables

**Figure 1:**
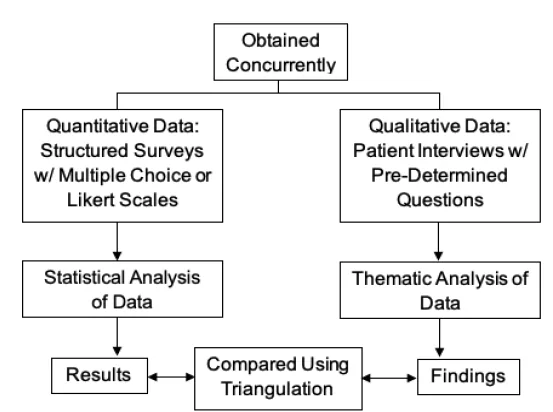
Convergent mixed-methods design framework in a study of community members’ expectations and concerns regarding a new emergency medicine training program.

**Figure 2:**
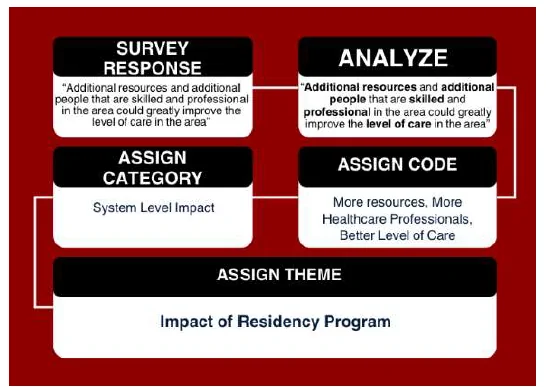
Thematic analysis methodology in a study of community members’ expectations and concerns regarding a new emergency medicine training program.

**Table:** Qualitative thematic analysis codebook: themes, categories, and codes.

Theme	Category	Code (*n* = times mentioned)
Benefits of Residency Program	System Level	Extra staff/Increased number of clinicians (26), Reduced wait times (4), Increased retention (11), Up-to-date training (8)
	Community Level	Economic benefit (1), Community benefit (5), Increased educatioal ppportunities (5)
Concerns Regarding Residents	Inexperience	Lack of knowledge (6), Rushing (3)
	Overconfidence	Arrogance (3), Not asking for help (10)
	Supervision	Lack of oversight (9), Proper supervision (3)
Trust (or Distrust)	Patient-Clinician Communication	Honesty (3), Professionalism (2), Bedside manner (8)
	Clinical Demeanor	Positive attitude (5), Listening skills (17)
Advice to Residentsfrom a Patient Perspective	Collaboration	Learn from staff (6)
	Preparedness	Stay updated (2), Humility (4), Knowledge (14)
