# Supplementary material for: Anticipating Change: Local Attitudes Toward a New Community Emergency Medicine Residency Program
Source: West J Emerg Med. 2026 Nov 2;27(6):1744–50. doi: 10.5811/westjem.65682 (PMC13761769; doi:10.5811/westjem.65682)
Supplement: Supplementary file 1 [file wjem-27-1744-s001.pdf]

Community Attitudes Toward Incoming Residency Programs at IU Health Bloomington

You are being invited to participate in a research study examining community members' experience with and perceptions of residency training programs. The study is being conducted by Dr. J. Austin Lee, MD MPH, an emergency medicine physician at the Indiana University School of Medicine.

Purpose of the Study

This study aims to explore community members' attitudes, expectations, and concerns regarding the upcoming introduction of resident physicians in the Emergency Department (ED) at IU Health Bloomington. The goal is to gather insights that will inform program planning, patient communication, and community engagement strategies ahead of the residency launch in 2026.

Number of People that Will Participate in this Study

We plan to collect data and interview several dozen community members to obtain their perspectives and experiences.

Procedures for the Study:

If you agree to participate in this study, you will participate in the following:
a 15-minute survey and brief recorded interview

RISKS OF PARTICIPATING IN THE STUDY:

While taking part in the study, the risks are considered to be minimal. Your participation will not delay your care in the Emergency Department. At any point you can tell the researcher that you are uncomfortable, do not care to answer a particular question, or no longer want to participate. Additionally, your voice will be recorded. We will not plan to collect any personally identifiable information, but if an unintended data breach were to occur a recording of your voice could potentially be accessed by individuals outside of the researcher team. We will take all possible steps to hope to reduce this possibility.

BENEFITS OF PARTICIPATING IN THE STUDY:

You will not directly benefit from this research.

CONFIDENTIALITY

Efforts will be made to keep your personal information confidential. All reports and transcripts will be held in confidence. The research team will be the only individuals who can access the recorded data. The recordings will be immediately transferred to a password protected computer. These recordings will then be removed from the recording

device and stored in a password-protected computer that only the research team will have access to. Audio recordings will be transcribed. After they have been transcribed, the recordings will be deleted. Transcripts of audio files and all other sources of data will be stored electronically.

PAYMENT

You will not be paid for participating in this study.

CONTACT FOR QUESTIONS OR PROBLEMS

If you have any questions about the study, contact J. Austin Lee, at 317-441-3711 or lee474@iu.edu

VOLUNTARY NATURE STUDY

Participation in this study is voluntary. You can choose not to participate at any time. Leaving the study will not result in any penalty or loss of benefits to which you are entitled. Your decision to participate or not in this study will not affect your current or future with your community or your community organization relationships.

CONSENT

Do you agree to participate in this study?

- ○ Yes
- ○ No

By selecting "YES", I certify that I am at least 18 years old.

- ○ Yes
- ○ No

By selecting "NO", I certify that I am not currently in the custody of the police, nor am I a prisoner.

- ○ Yes
- ○ No

Awareness

How would you describe an emergency medicine resident?
* must provide value

- ◯ College student interested in becoming an emergency medicine physician
- ◯ Medical student interested in becoming an emergency medicine physician
- ◯ Someone who graduated from medical school, in supervised training to specialize in emergency medicine
- ◯ Someone who has completed medical school and is ready to practice independently
- ◯ I don't know
- ◯ None of the above

reset

I am aware that IUHB will have a resident training program in EM in 2026.
* must provide value

- ◯ Yes
- ◯ No

reset

I have received care from a resident physician before (at IU Health or elsewhere).
* must provide value

- ◯ Yes
- ◯ No

reset

What advice would you give to an incoming resident to improve their ability to provide care?

Benefits

Do you think there are potential benefits of training residents at IU Health - Bloomington? If so, what are

IU Health Bloomington will become a better hospital by training residents.
* must provide value

- ◯ Strongly agree
- ◯ Agree
- ◯ Neither agree nor disagree
- ◯ Disagree
- ◯ Strongly disagree

Training residents in the Bloomington community will improve the quality of health care.
* must provide value

- ◯ Strongly agree
- ◯ Agree
- ◯ Neither agree nor disagree
- ◯ Disagree
- ◯ Strongly disagree

Training residents will improve access to health care in Bloomington and surrounding areas.
* must provide value

- ◯ Strongly agree
- ◯ Agree
- ◯ Neither agree nor disagree
- ◯ Disagree
- ◯ Strongly disagree

Bloomington and surrounding areas have a shortage of healthcare providers.
* must provide value

- ◯ Strongly agree
- ◯ Agree
- ◯ Neither agree nor disagree
- ◯ Disagree
- ◯ Strongly disagree

Resident physicians can bring the most up-to-date medical knowledge and practices to patient care.
* must provide value

- ◯ Strongly agree
- ◯ Agree
- ◯ Neither agree nor disagree
- ◯ Disagree
- ◯ Strongly disagree

Concerns

What concerns you about receiving care from a resident physician?

I am concerned that having resident physicians involved in my care might negatively affect my treatment.
* must provide value

- ◯ Strongly agree
- ◯ Agree
- ◯ Neither agree nor disagree
- ◯ Disagree
- ◯ Strongly disagree

I do not feel comfortable with residents' level of training.
* must provide value

- ◯ Strongly agree
- ◯ Agree
- ◯ Neither agree nor disagree
- ◯ Disagree
- ◯ Strongly disagree

Trust

I trust future residents to provide quality health care.
* must provide value

- ◯ Strongly agree
- ◯ Agree
- ◯ Neither agree nor disagree
- ◯ Disagree
- ◯ Strongly disagree

I would be comfortable receiving care from a resident physician in the Emergency Medicine Department at IU Health - Bloomington.
* must provide value

- ◯ Strongly agree
- ◯ Agree
- ◯ Neither agree nor disagree
- ◯ Disagree
- ◯ Strongly disagree

I trust IU Health - Bloomington to ensure that resident physicians provide safe and effective care.
* must provide value

- ◯ Strongly agree
- ◯ Agree
- ◯ Neither agree nor disagree
- ◯ Disagree
- ◯ Strongly disagree

I would prefer to have a supervising physician present for the majority of my interactions with a resident.
* must provide value

- ◯ Strongly agree
- ◯ Agree
- ◯ Neither agree nor disagree
- ◯ Disagree
- ◯ Strongly disagree

What features of resident physicians lead you to trust/not trust them with your care?

Future

I believe residency programs will improve health care in the Bloomington and surrounding areas.

* must provide value

- ◯ Strongly agree
- ◯ Agree
- ◯ Neither agree nor disagree
- ◯ Disagree
- ◯ Strongly disagree

I would support the expansion of other residency or medical training programs in the future.

* must provide value

- ◯ Strongly agree
- ◯ Agree
- ◯ Neither agree nor disagree
- ◯ Disagree
- ◯ Strongly disagree

Why do you think expanding residency/medical training programs WILL improve health care in the Bloomington and surrounding areas?
