# Supplementary material for: Anticipating Change: Local Attitudes Toward a New Community Emergency Medicine Residency Program
Source: West J Emerg Med. 2026 Nov 2;27(6):1744–50. doi: 10.5811/westjem.65682 (PMC13761769; doi:10.5811/westjem.65682)
Supplement: Supplementary file 2 [file wjem-27-1744-s002.pdf]

Anticipating Change: Community Attitudes Toward Incoming Residency Programs at IU Health Bloomington

Purpose of the Study

This study aims to explore community members' attitudes, expectations, and concerns regarding the upcoming introduction of resident physicians in the Emergency Department (ED) at IU Health Bloomington. The goal is to gather insights that will inform program planning, patient communication, and community engagement strategies ahead of the residency launch in 2026.

Who We Anticipate Interviewing and Why We Chose Them

We plan to collect data and interview several dozen community members to obtain their perspectives and experiences. Patients will be recruited in a convenience sample method in the IU Health Bloomington ED. Patients who are either in the waiting room or in a clinical care space will be screened based on their age and other eligibility criteria. We chose to interview these patients because they are relevant to the study (being in the ED), easy to access without interfering with care, and are a diverse group of community members.

Pre-Survey Script

"Hi my name is Ben Johnson, and I am a medical student conducting research here at the hospital. There are two new residency programs coming to IU Health - Bloomington in 2026. We would like to know what the community thinks about these new programs and use that information to improve community outreach and resident training practices. Would you be willing to participate in a 15-minute survey to ask your thoughts on these new programs?"

If yes: "This survey is part of a research project to gather input from the community to inform new residency programs coming to Bloomington. By participating in the survey, you would help us better understand community perceptions of the new programs. Your participation will not delay your care here today. Your participation is voluntary, and we will not collect any sensitive information such as your name, DOB, or any medical information. We will plan to record your voice for a few of the questions to help better analyze responses. If you have any questions or concerns, feel free to contact the lead researcher, Dr Lee. Do you consent to take part in this research?

Anticipating Change Survey Protocol

Residency Program Awareness:

Follow-up Questions:
1) What did the resident do that made the experience favorable/unfavorable?
2) What advice would you give to an incoming resident to improve their ability to provide care?

Potential Benefits of Residency Programs

Follow-up Question:
1) Do you think there are potential benefits of training residents at IU Health - Bloomington? If so, why?

Potential Concerns with Residency Programs

Potential Follow-up Question:
1) What concerns you about receiving care from a resident physician?

Resident Physicians and Trust

Follow-up Question:
1) What features of resident physicians lead you to trust/not trust them with your care?

Looking to the Future of Emergency Medicine at IU Health – Bloomington

Follow-up Question:
1) Why do you think expanding residency/medical training programs will improve/not improve health care?
