# Supplementary material for: Anticipating Change: Local Attitudes Toward a New Community Emergency Medicine Residency Program
Source: West J Emerg Med. 2026 Nov 2;27(6):1744–50. doi: 10.5811/westjem.65682 (PMC13761769; doi:10.5811/westjem.65682)
Supplement: Supplementary file 3 [file wjem-27-1744-s003.pdf]

Question		
**Answers**	**N**	**%**
How would you describe an emergency medicine resident?		
College student interested in becoming an emergency medicine physician	4	10.3%
Medical student interested in becoming an emergency medicine physician	9	23.1%
Someone who graduated from medical school, in supervised training to specialize in emergency medicine	19	48.7%
Someone who has completed medical school and is ready to practice independently	3	7.7%
I don’t know	4	10.3%
None of the above	0	0.0%
I am aware that IUHB will have a resident training program in EM in 2026		
No	16	41.0%
Yes	23	59.0%
I have received care from a resident physician before (at IU Health or elsewhere)		
No	19	48.7%
Yes	20	51.3%

If yes, I was satisfied with the care that I received from the resident physician		
Strongly Agree	8	40.0%
Agree	11	55.0%
Neither Agree/Disagree	1	5.0%
Disagree	0	0.0%
Strongly Disagree	0	0.0%
**IU Health Bloomington will become a better hospital by training residents**		
Strongly Agree	27	69.2%
Agree	10	25.6%
Neither Agree/Disagree	2	5.1%
Disagree	0	0.0%
Strongly Disagree	0	0.0%
**Training residents in the Bloomington community will improve the quality of health care**		
Strongly Agree	24	61.5%
Agree	13	33.3%
Neither Agree/Disagree	2	5.1%
Disagree	0	0.0%
Strongly Disagree	0	0.0%

Training residents will improve access to health care in Bloomington and surrounding areas		
Strongly Agree	25	64.1%
Agree	13	33.3%
Neither Agree/Disagree	1	2.6%
Disagree	0	0.0%
Strongly Disagree	0	0.0%
**Bloomington and surrounding areas have a shortage of healthcare providers**		
Strongly Agree	14	35.9%
Agree	16	41.0%
Neither Agree/Disagree	8	20.5%
Disagree	1	2.6%
Strongly Disagree	0	0.0%
**Resident physicians can bring the most up-to-date medical knowledge and practices to patient care**		
Strongly Agree	15	38.5%
Agree	22	56.4%
Neither Agree/Disagree	2	5.1%
Disagree	0	0.0%

Strongly Disagree	0	0.0%
I am concerned that having resident physicians involved in my care might negatively affect my treatment		
Strongly Agree	0	0.0%
Agree	2	5.1%
Neither Agree/Disagree	9	23.1%
Disagree	21	53.8%
Strongly Disagree	7	17.9%
I do not feel comfortable with residents' level of training		
Strongly Agree	0	0.0%
Agree	1	2.6%
Neither Agree/Disagree	7	17.9%
Disagree	21	53.8%
Strongly Disagree	10	25.6%
I trust future residents to provide quality health care		
Strongly Agree	10	25.6%
Agree	26	66.7%
Neither Agree/Disagree	3	7.7%

Disagree	0	0.0%
Strongly Disagree	0	0.0%
I would be comfortable receiving care from a resident physician in the Emergency Medicine Department at IU Health - Bloomington		
Strongly Agree	10	25.6%
Agree	24	61.5%
Neither Agree/Disagree	4	10.3%
Disagree	1	2.6%
Strongly Disagree	0	0.0%
I trust IU Health - Bloomington to ensure that resident physicians provide safe and effective care		
Strongly Agree	13	33.3%
Agree	23	59.0%
Neither Agree/Disagree	2	5.1%
Disagree	1	2.6%
Strongly Disagree	0	0.0%
I would prefer to have a supervising physician present for the majority of my interactions with a resident		
Strongly Agree	4	10.3%

Agree	18	46.2%
Neither Agree/Disagree	14	35.9%
Disagree	3	7.7%
Strongly Disagree	0	0.0%
I believe residency programs will improve health care in the Bloomington and surrounding areas		
Strongly Agree	12	30.8%
Agree	25	64.1%
Neither Agree/Disagree	2	5.1%
Disagree	0	0.0%
Strongly Disagree	0	0.0%
I would support the expansion of other residency or medical training programs in the future		
Strongly Agree	15	38.5%
Agree	23	59.0%
Neither Agree/Disagree	1	2.6%
Disagree	0	0.0%
Strongly Disagree	0	0.0%
